# Antenatal CFTR Modulators to Treat a Healthy Pregnant Woman with a Fetus Affected by Cystic Fibrosis Complicated by Meconium Ileus and Intestinal Volvulus: From a Suspicion of the Disease to a Targeted Treatment in Utero: Case Report and Narrative Review

**DOI:** 10.3390/jcm15051933

**Published:** 2026-03-04

**Authors:** Ramona Montironi, Stefano Raffaele Giannubilo, Irene Cappanera, Giulia Capogrosso, Romina Mancinelli, Arianna Rinci, Andrea Ciavattini

**Affiliations:** Department of Clinical Sciences, Obstetrics and Gynecology Section, Università Politecnica delle Marche, Via Filippo Corridoni 11, 60123 Ancona, Italy; ramonamontironi@gmail.com (R.M.); irene.cappanera@gmail.com (I.C.); giuliagiuggi94@gmail.com (G.C.); romina.mancinelli@ospedaliriuniti.marche.it (R.M.); arianna.rinci@ospedaliriuniti.marche.it (A.R.); a.ciavattini@univpm.it (A.C.)

**Keywords:** fetus, cystic fibrosis, meconium ileus, intestinal volvulus, CFTR, ETI

## Abstract

The introduction of cystic fibrosis transmembrane conductance regulator modulators (CFTRms) in the treatment of cystic fibrosis (CF) has significantly improved both the life expectancy and quality of life for patients affected by the disease. While this new therapy shows promising results in CF patients, data about the use of CFTRms during pregnancy in women who are carriers with fetuses affected by CF are limited. We present a new case report and literature review of fetal CF treated by the maternal assumption of CFTRms to understand the safety and the effectiveness of the treatment in resolving fetal and neonatal CF clinical manifestations (meconium ileus, postnatal exocrine pancreatic insufficiency, and postnatal intestinal surgery). Our report represents the fifth unsuccessful case of fetal CF treated in utero by CFTRms and highlights the potential key elements necessary for a successful treatment.

## 1. Introduction

The advent of cystic fibrosis transmembrane conductance regulator modulators (CFTRms) has significantly increased the median life expectancy and dramatically improved the quality of life of patients affected by cystic fibrosis (CF). Consequently, more women reach the childbearing age, and clinicians are urgently looking for data about the use of CFTRms during pregnancy and breastfeeding, especially regarding their effectiveness and safety for both mother and fetus. CFTRms improve pancreatic and lung function with greater benefits the earlier treatment begins. That is why the triple combination of drugs Elexacaftor/tezacaftor/ivacaftor (ETI) was initially approved by the U.S. Food and Drug Administration in 2019 for patients 12 years of age and older with at least one F508del variant. Then, in November 2023, the European Commission expanded the indication for KAFTRIO^®^ (ivacaftor/tezacaftor/elexacaftor) (Vertex Pharmaceuticals, London, UK) in a combination regimen with ivacaftor to treat patients aged 2 years and older and at least one F508del mutation. Italy’s drug agency (AIFA) approved the extension of this therapeutic indication in July 2025. However, KAFTRIO^®^ is not officially licensed for use in pregnancy worldwide due to limited human data. To date, data on using CFTRms in the fetus are still scarce, but there is evidence that CFTRms administered during pregnancy have no teratogenic effects, cross the placenta, and pass in breast milk, so that the fetus and the newborn can be treated too [1,2,3,4,5]. These findings were initially suggested by a revolutionary report by Fortner et al. They described a mother affected by CF who was elected to remain on modulator therapy to maintain her health during her pregnancy. She delivered a newborn with CF who had pancreatic sufficiency and a false negative CF screen, suggesting transplacental treatment could have beneficial effects for the fetus [6]. Recent reports and series have demonstrated the resolution of meconium ileus (MI) in most CF fetuses treated with transplacental CFTRms, but not in all cases [7,8,9,10,11,12]. We present a new case report along with a literature review on fetal CF treated through maternal administration of CFTRms with the following objectives: (1) to assess the safety and the effectiveness of treatment in resolving fetal and neonatal CF clinical expressions: prenatal MI, postnatal exocrine pancreatic insufficiency (EPI), and postnatal intestinal surgery; (2) to identify what factors influence the success of in utero-therapy.

## 2. Case Report

**Figure 1:**
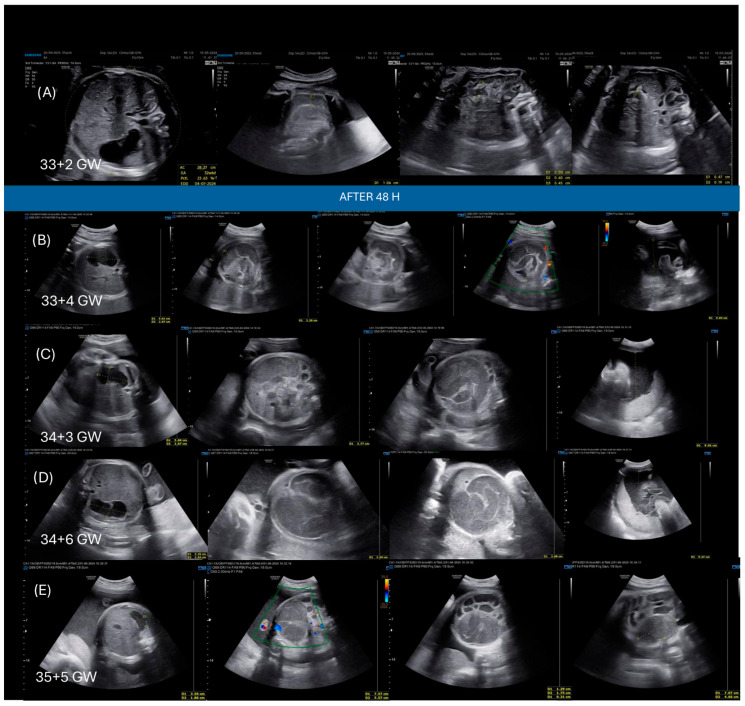
The sonographic evolution of meconium ileus complicated by intestinal volvulus in a fetus affected by cystic fibrosis. (**A**) Bowel dilatation of a maximum of 11 mm at 33 + 2 gestational weeks (GW). (**B**–**D**) Appearance of significant bowel dilatation with a spiral course of the loops dilated (“whirlpool sign”) with progressive increase in dilatation and appearance of polyhydramnios. (**E**) Appearance of meconium pseudocyst.

A 31-year-old Caucasian woman, G2P1, was referred to our institution at 22 gestational weeks (GW) because of the presence of moderate fetal bowel hyperechogenicity and dilatation at the routine second-trimester ultrasound (US). Her previous pregnancy was uneventful, and the medical history of the couple and their families was negative for relevant illnesses. However, the current pregnancy revealed at the reference scan a fetus with moderate bowel hyperechogenicity and an apparent absence of the gallbladder. No significant intestinal dilatation and other fetal anomalies were noted, and the fetal growth was normal. Cell-free DNA (cfDNA) resulted in a low risk for fetal aneuploidy. For these reasons, the patient was counseled to perform TORCH complex, parental screening for CF, and a magnetic resonance of the fetus. TORCH complex was negative, excluding an infectious cause of bowel hyperechogenicity. The magnetic resonance confirmed the suspicion of the absence of the gallbladder and the absence of significant bowel dilatations. Whereas the parental screening for CF revealed a heterozygote state of the F508del mutation for both parents. Thus, a genetic characterization of the fetus was performed through an amniocentesis at 31 GW and revealed a fetus affected by CF with a homozygote F508del genotype. The couple decided to continue the pregnancy; hence, a multidisciplinary team (cystic fibrosis unit, obstetricians, neonatologists, and pediatric surgeons) evaluated the possibility of maternal therapy with ETI to mitigate CF-related complications in the fetus. It was ultimately decided not to initiate ETI treatment but rather to monitor the fetus closely with US examinations every two weeks, given the lack of significant bowel dilatation. At 33 + 2 GW, the US revealed no substantial modifications in the gastrointestinal tract. The bowel dilatation was a maximum of 11 mm only in a short tract, the stomach dimensions were at the upper limit, the amniotic fluid was normal, and the fetal growth was regular for the gestational age (Figure 1A). Thus, a new scan was scheduled for 2 weeks later. However, 48 h after the last scan (33 + 4 GW), the mother returned to the hospital because of a reduction in fetal movements during the night. The US revealed significant changes in the intestinal tract: the bowel showed a dilatation of 22 mm with a spiral course of the dilated loops. Ascites was not present, amniotic fluid was still regular, and cardiotocography (CTG) was normal despite poor variability (Figure 1B). Given the rapid deterioration of the intestinal findings, the reduction in fetal movements, and the gestational age, the patient was hospitalized with the suspicion of acute intestinal obstruction due to MI and doubtful presence of intestinal volvulus. At admission, after a new multidisciplinary discussion, the patient received betamethasone to prevent respiratory distress syndrome (RDS) and started ETI treatment to try to resolve the MI with Kaftrio^®^ (75/50/100 mg, 2 oral tablets in the morning) and Kalydeco^®^ (Vertex Pharmaceuticals Dublin, Ireland) (150 mg, 1 oral tablet in the evening). Throughout her hospitalization, the mother continued ETI treatment for a total of 21 days without any side effects. Her liver function remained normal, and she reported a gradual improvement in fetal movement perception as well as stable CTG results. Only a mild skin rash appeared on the first day of treatment, which resolved spontaneously within a few hours. In contrast, the fetus exhibited a progressive worsening of sonographic findings. The bowel continued to dilate, maintaining its spiral course and reaching a diameter of 30 mm at 34 + 6 GW. Polyhydramnios progressively developed, and the stomach remained moderately dilated (Figure 1C,D). At 35 + 5 GW, the US findings changed again: the stomach returned to normal dimensions, bowel dilatation returned at 11–12 mm with hyperechoic and thick walls, and a meconium-filled cyst with a thick membrane of 7.5 × 5.5 × 4.6 cm appeared, always in the absence of ascites (Figure 1E). Despite concerns regarding intestinal perforation due to the meconium pseudocyst, it was determined to continue therapy and manage the situation expectantly, given the normal CTG readings and stable perception of fetal movements. An urgent cesarean section was conducted a few days later at 36 + 4 GW because of preterm premature rupture of membranes and breech presentation. A 2920 g male neonate was born with Apgar scores of 8 and 8 at 1 and 5 min, respectively. The newborn required no resuscitation and was admitted to the neonatal intensive care unit (NICU) due to bowel obstruction and mild prematurity. A chest radiograph showed no abnormalities, but postnatal clinical and radiological abdominal examinations confirmed the antenatal findings. The abdomen was distended with a palpable mass in the right and central abdominal quadrants. The nasogastric tube recovered yellowish material, and no passage of meconium was observed. Supine abdominal radiograph and ultrasonogram verified the presence of a large cyst in the central part of the abdomen of 7.5 × 5. 5 cm, mild jejunal dilatation with some air–fluid levels, microcolon, and no passage of rectal Gastrograffin over distal ileus. A laparotomy was performed 12 h after birth. Surgical findings confirmed a MI complicated by a jejuno volvulus (three turns around the mesenteric axis) and intestinal perforation with fibroadhesive peritonitis and a meconium pseudocyst located in front of the point of intestinal perforation. A resection of the perforated and dilated bowel loop, manual emptying of the dehydrated thickened meconium, and a temporary terminal ileostomy (55 cm distal to the ligament of Treitz and 70 cm proximal to the ileocecal valve) were performed. Postnatal laboratory values revealed a cholestasis and a severe EPI: fecal pancreatic elastase levels at 2 and 21 days of life (DOL) was <15 mcg/g (normal > 200 mcg/g), total bilirubin at DOL1 was 2.68 mg/dL (normal < 1.20 mg/dL), direct bilirubin at DOL 1 was 0.83 mg/dL (normal < 0.30 mg/dL), gamma-glutamyl transferase (GGT) at DOL 1 was 296 U/L (normal ≤ 73 U/L). The newborn had exclusive parenteral nutrition (PN) during the first days after surgery. He started pancreatic enzyme replacement therapy (PERT) and minimal enteral feeding (MEF) (5 mL/kg/day) using breastmilk and medium-chain triglyceride (MCT) oil from DOL 6. On DOL 6, the baby reached the maximum weight loss (−17% of the birth weight), with a progressive weight gain in the days that followed. At DOL 27, an episode of late-onset central venous catheter (CVC)-related sepsis due to candida albicans was treated with targeted antifungal therapy. The day after, the baby started 4-h cycles of Continuous Positive Airway Pressure (CPAP)/day, associated with respiratory physiokinesitherapy to prevent atelectasis. The Ileo-ileal anastomosis was performed at DOL 48, and full enteral feeding (FEF) was reached at DOL 60. The baby was discharged home on DOL 63, weighing 3684 Kg and nutrition composed of human milk in association with PERT, MCT oil, and sodium chloride (NaCl) supplementation.

## 3. Literature Review

We examine the literature by searching scientific articles in PubMed/MEDLINE, Scopus, and ScienceDirect medical databases. We used the search terms “fetal cystic fibrosis, treatment transmembrane” as keywords from inception to 18 June 2025. The inclusion criteria consider all human cases of fetal cystic fibrosis treated in utero by the maternal assumption of CFTRm therapy. Exclusion criteria were all animal cases of fetal cystic fibrosis and cases with no full text available. A total of 165 articles (89 in PubMed/MEDLINE, 66 in Scopus, and 10 in ScienceDirect) matched the keywords “fetal cystic fibrosis, treatment transmembrane”. The authors (R.M., I.C., and G.C.) analyzed the titles and abstracts of these articles to determine which could undergo full-text review. This analysis excluded 159 articles based on their titles and abstracts, and 6 papers were independently identified and met the inclusion criteria after full-text evaluation.

With our report, 21 CF fetuses treated in utero by the maternal assumption of CFTRms had been reported in the literature until June 2025. All data are from case reports and series and are detailed in Table S1 (Supplementary Material). One fetus has to be excluded because the mother declined ETI once the indication was switched from curative to preventative. All 20 fetuses had at least one F508del mutation: 12 were homozygous for F508del, and 8 were heterozygous. These mutations were diagnosed through amniocentesis, chorionic villus sampling, and, in one case, non-invasive prenatal diagnosis. The indication for treatment was curative in 16 cases, due to US findings suggestive of MI (12/16) or MI complications (4/16), and preventative in 4 cases (tertiary prevention of prenatal CF-related damage). A total of 19 fetuses were treated by ETI and one with ivacaftor alone, from 19–36 GW until birth. However, one fetus only received the first dose of ETI because the mother chose to terminate the pregnancy due to a suspicion of fetal intestinal volvulus. Another fetus received only two doses of ETI as it was born 36 h after treatment initiation. Additionally, one fetus stopped ETI due to a pruritic eruption on day 15. Only 6 of the 20 cases continued ETI during breastfeeding. In all cases, no teratogenic effects were observed. Preterm birth occurred in 4/20 cases, and only 1 case had fetal growth restriction. All the mothers demonstrated good tolerance to the treatment, with only 3/20 cases experiencing a mild skin rash and no further complications. All newborns presented with EPI and required PERT, except for baby 18 (who carried a mutation associated with pancreatic sufficiency) and baby 4 (for whom no data were available). The absence of the vas deferens was found in 2/7 of the baby boys. Furthermore, 3/19 neonates had cholestasis, 2/19 had elevated gamma-glutamyl transferase (GGT) levels, and 1/19 had unconjugated hyperbilirubinemia. We emphasize that transaminase elevation is a very common side effect of ETI, so it is not necessarily a consequence of the CF alone. In the preventative group, fetal US was normal throughout the pregnancy, and no intestinal obstruction was observed after birth in all 4 cases. In the curative group, ETI resolved antenatal MI, thus preventing postnatal intestinal obstruction and surgery in most cases (10/16). However, ETI failed to resolve MI in 5 out of 16 cases, leading to postnatal surgery in 4 of those cases.

## 4. Discussion

The discovery of the CFTR gene in 1989 has progressively changed the landscape of CF management and research. It has led to the identification of more than 2000 mutations related to CF and the development of new therapeutic strategies targeting the dysfunctions caused by specific CFTR mutations. In fact, since 2012, the approach to CF treatment has translated from “symptomatic” (focusing mainly on slowing lung disease progression, intestinal malabsorption, and compensation of pancreatic insufficiency) to “therapeutic” with the advent of new drugs called CFTRms. This advancement has increased the life expectancy from early childhood in the 1960s to 40–50 years nowadays [13]. Specifically, CFTRms are small, orally administered molecules that target defective CFTR proteins, improving their production, intracellular processing, and/or function. If given early, they have the potential to slow down the progression of the disease. However, their indications and efficacy depend on the CFTR mutations of the individual patient. The most widely used modulator is KAFTRIO^®^ (ivacaftor/tezacaftor/elexacaftor) in a combination regimen with ivacaftor, as it is approved for patients aged 2 years and older with at least one F508del mutation (the most common mutation found in approximately 80% of people with CF) [14,15]. ETI has been shown to be effective in improving lung function, reducing the rates of respiratory exacerbations, increasing weight, and improving quality of life in young children, adolescents, and adults [16,17,18,19]. However, their efficacy on the extrapulmonary manifestations of CF is still less established, as well as their effects on the disease when administered in pregnancy, breastfeeding, and newborns [20]. These data should not be understated, as patients with CF are living longer and more people are reaching the childbearing age. Moreover, if respiratory diseases remain the leading cause of morbidity and mortality in CF children and adults, gastrointestinal complications are the main manifestations during intrauterine life. The new challenge lies in understanding the efficacy and the safety of CFTRms for both the CF mother and the CF fetus and how to monitor these pregnancies, especially considering that ETI can cross the placenta and can be detected in breast milk [1,2,3]. In the fetus and the neonate, the earliest manifestations of CF are MI and EPI [21], with MI frequently being the initial sign of the disease. MI is a small bowel obstruction caused by thick, sticky meconium that typically involves the terminal ileum. It initially develops in utero and occurs in 12.5–25.9% of newborns with CF, especially those with class I-III CFTR mutations (typically associated with lower CFTR function) [22,23]. MI can be suspected on antenatal US when the bowel is hyperechoic and/or dilated; however, its antenatal findings can differ in complex forms. Approximately 50% of MIs are complicated by segmental volvulus, intestinal atresia, ischemic necrosis, or perforation [24]. When perforation occurs, it can lead to meconium peritonitis (resulting from the extrusion of meconium into the abdominal cavity) or pseudocyst formation (resulting from the encapsulation of meconium within an intestinal membrane) [25]. These complex MIs require urgent surgical treatment after birth, with long-term gastrointestinal issues and increased hospital costs. EPI is also an early manifestation of CF and results from the blockage of sticky pancreatic secretions in the pancreatic ducts, with a profound impact on nutrition and growth. It also begins in utero and affects about 60% of newborns with CF [26,27]. Unlike MI, to date, there are no antenatal sonographic findings suggestive of EPI in utero. Based on our report and research, transplacental ETI seems to be safe and effective in resolving prenatal MI in most cases. However, it showed no efficacy in preventing postnatal EPI. Our case is the fifth where ETI did not resolve MI and became complicated by intestinal volvulus, subsequent perforation, and formation of a giant meconium pseudocyst. These antenatal intestinal complications had multiple consequences in the neonate since the baby had intestinal resection 12 h after birth, an ileo-ileal anastomosis after 48 days, a CVC–related sepsis, a FEF reached after 60 days, and a total of 63 days of hospitalization. In light of the collected data, we suggest that the reasons for this failure may be related to the gestational age and the severity of the intestinal US findings at the onset of treatment. The 10 resolved cases started maternal ETI between 26–34 GW (averaging at 30 GW) with a fetal bowel dilatation of a maximum of 11–12 mm, requiring 3 to 56 days (average of 26 days) of therapy to resolve MI. Whereas in the 5 unresolved cases (all F508del homozygous), the mother initiated ETI between 27–36 GW (averaging 32 GW) when the fetal bowel had a diameter of at least 14 mm, and in most cases, showed already signs of MI complications (3/5). These US findings are not surprising, as the fetal bowel reaches its highest functional maturity at the beginning of the third trimester, resulting in a peak incidence of intestinal volvulus, perforation, and meconium peritonitis/pseudocyst, especially after 30 GW [28,29,30]. Furthermore, the mother’s body weight may also influence the effectiveness of ETI, as raised by case 4: the mother weighed 170 kg, and ETI failed to resolve MI despite being started at 27 GW, albeit with a bowel dilation of 14 mm [10]. The role of the total duration of therapy on the effectiveness of ETI remains doubtful but is a reasonably critical element to consider. The cases of Fortner et al. and Kowalik et al. [6,31] (not eligible for our research strategy) represent the only fetuses with homozygous F508del mutation who received ETI throughout the pregnancy, showing no signs of MI and postnatal EPI. These data suggest the possible efficacy of transplacental ETI on preventing (rather than resolving) fetal MI and postnatal EPI when administered from the beginning of pregnancy.

## 5. Conclusions

ETI is an innovative treatment not only for children and adults but also for fetuses affected by CF. When administered via carrier healthy mother to treat CF fetus, it seems safe and promising in resolving MI and preventing its complications (intestinal volvulus, necrosis, perforation, and meconium peritonitis/pseudocyst). This is extremely important since gastrointestinal diseases are often the earliest manifestation of CF and contribute to significant neonatal morbidity and mortality, especially in patients with more severe CFTR mutations (class I–II–III). Using ETI can avoid postnatal intestinal surgery and its impact on parenteral nutrition dependency and duration, neonatal growth as well as longer hospital admissions. However, the gestational age and the severity of the intestinal condition at the beginning of treatment seem to play a crucial role in the success of the therapy in utero. This paper highlights this concept for the first time. Our report is the fifth to demonstrate that ETI has no beneficial effects in resolving antenatal MI, as it was initiated at 34 GW and when MI was already complicated due to a sudden change in intestinal findings. Therefore, we believe that transplacental ETI should be started at the first signs of MI and ideally not over 30 GW, when there is the highest risk of MI complications. Further in-utero ETI cases are encouraged to be published to confirm gestational age and US intestinal findings at the beginning of treatment as critical elements in resolving fetal MI. Additionally, we aim to understand if ETI can play a role in the prevention of prenatal MI and postnatal EPI when started shortly after a fetal CF diagnosis. Moreover, additional cases are essential to enhance our understanding of ETI’s passage across the placenta and in breast milk, as well as to determine if individualized dosages are necessary, particularly for individuals with higher body weights.

## Data Availability

Data sharing not applicable to this article as no datasets were generated or analyzed during the current study.

## Supplementary Materials

The following supporting information can be downloaded at: https://www.mdpi.com/article/10.3390/jcm15051933/s1.
