# Supplementary material for: Antenatal CFTR Modulators to Treat a Healthy Pregnant Woman with a Fetus Affected by Cystic Fibrosis Complicated by Meconium Ileus and Intestinal Volvulus: From a Suspicion of the Disease to a Targeted Treatment in Utero: Case Report and Narrative Review
Source: J Clin Med. 2026 Mar 4;15(5):1933. doi: 10.3390/jcm15051933 (PMC12985462; doi:10.3390/jcm15051933)
Supplement: Supplementary file 1 [file jcm-15-01933-s001.zip › jcm-4058758-supplementary.pdf]

Table S1. Cases of fetal cystic fibrosis treated in utero by maternal assumption of CFTR modulators (Review of Literature until 18 June 2025.

Authors (Y)	Sp size	n°	Fetal genotype	CFTRm	CFTRm initiation (GW)	Fetal US findings	CFTRm initiation to resolution of US findings (D)	GA at birth (W)	Sex/ BW (g)	Postnatal presentation	CFTRm during breast feeding	Postnatal surgery	Postanatal treatment
**Szentpetery S. et al. 2022 [7]**	1	1	F508del/ F508del	ETI	32	23 gw→ single loop of BD<br>28 gw→ BH, BD (11),GB(nd)<br>33 gw → BH, BD(14)GB(nd)	27	36	F/2280	*EPI*<br>(No BO)	Yes	No	*PERT from*<br>DOL 1.<br>Discharged home on DOL2
**Gomez-Montes E. et al. 2023 [8]**	1	2	F508del/ F508del	ETI	31	21 gw→ BH, GB(nd)<br>25 gw→ BH, BD(12.6),<br>33gw→ BH, BD (10-11),<br>mild ascites, GB(nd)	56	39	M/ 2750	*EPI*<br>(No BO)	Yes	No	*PERT* from DOL 10<br>Discharged home before DOL10
**Blumenfeld YJ. et al. 2023 [9]**	1	3	F508del/ F508del	ETI	26	23 gw→ BH, BD, GB(nd)<br>29 gw→MI and distal microcolon at MRI, GB(nd)	49	39	F/2850	*EPI*<br>(No BO)	ND	No	PERT<br>Discharged home on DOL 4
**Destoop M. et al. 2025 [11]**	1	4	F508del/ F508del	ETI	27	21gw →BH with pathological dilation, GB(nd)<br>25+6 gw → BD (9.4), GB(nd)<br>27 gw → BD (14), GB(nd)<br>33 gw→ BD (25), GB(nd)<br>35 gw → BD (30), GB(nd)<br>37+6gw→BD (27), GB(nd)	No resolution	39	ND/ Nl weight	*BO*<br>*(* MI and severe microcolon)	Yes<br>(DOL7-28)	Yes	Emergency ileostomy on DOL 1<br>FEF reached on DOL 7<br>PERT ND
**Metcalf A. et al. 2025 [10]**	3	5	F508del/ F508del	ETI	31	BH from 17 gw, GB(nd)	56	39	F/ND	*EPI*<br>(No BO)	Stopped on DOL 4	No	PERT
		6	F508del/ F508del	ETI	30	20gw →BH, GB(nd)<br>28w →BH , BD, GB(nd)<br>36gw→ multiple BD, GB(nd)	No resolution	37	F/ND	*EPI*<br>Persistence of *MI*	Yes	No	Rectal irrigation on DOL 1-2, PERT
		7	F508del/ F508del	ETI	35	28 gw →BH, BD, GB(nd)<br>33 gw → jejunal atresia, BD, meconium preudocyst on MRI, GB(nd)	No resolution	38	F/ND	*BO*<br>(MI,<br>jejunal atresia)<br>*EPI*<br>*Mild cholestasis*	No	Yes	LPT: removal of meconium plugs, jejunal atresia repair and jejunostomy with minimal bowel resection<br>PERT after restoration enteral feedings<br>Jejuno-jejuno anastmosis (DOL 42)
**Bonnel AS. et al. 2025 [12]**	13	8	F508del/ F508del	ETI	34	27gw→BH;BD(no);GB(nd)<br>34gw→BH; BD(9);GB(nd)	5	35	M/ 3390	*EPI*<br>(No BO)<br>*Vas deferens: NF*	No	No	PERT
		9	F508del/F508del	ETI	30	24 gw→BH; BD(15)<br>30 gw→suspicion of intestinal volvulus on MRI	VTP after the first ETI intake	30	M/ND	*VTP*<br>*(MI,* intestinal volvulus)	-	-	-
		10	F508del/ F508del	ETI	36	32 gw→BH (no); BD(no); GB(yes); ascites; hepatosplenomegaly<br>36 gw→ BH (no); BD(no); GB(yes); ascites; hepatosplenomegaly	No resolution, only 2 doses exposed (born after 36 h)	36	M/ 2880	*EPI*<br>*BO*<br>*(* MI, meconial peritonitis, bowel perforation)<br>*Neurodegenerative disese* (2 months)	No	Yes	PERT<br>Surgery on DOL2

		11	c2959–2960dup/ F508del	ETI	31	24gw→BH; BD(10); GB (yes)<br>30gw→BH; BD(no); GB (no)	Stopped ETI at d 15 for pruriginous eruption	38	M/ 3000	*EPI*<br>(No BO)	No	No	PERT
		12	F508del/ G452P	ETI	33	22 gw→BH; BD(no);GB (no)<br>32gw→BH; BD(no); GB (no)	15	37	M/ 3630	*EPI*<br>(No BO)<br>*Transient RDS*<br>*Vas deferens: NF*	No	No	PERT
		13	M1V/ F508del-Q1313K	ETI	34	19 gw→BH; BD(no); GB (nd)<br>32gw→BH; BD(12); GB (nd)	26	39	F/2320	*EPI*<br>(No BO)<br>*Cholestasis*	No	No	PERT<br>Ursodesoxycholic acid
		14	F508del/ F508del	ETI	28	22 gw→BH; BD(6); GB (yes)<br>27gw→BH; BD(9); GB (nd)	3	34	F/2320	*EPI*<br>(No BO)	No	No	PERT
		15	G542X/ F508del	NA	32	25gw→BD(10); GB (yes)<br>32→Nl MRI	Mother refusal initiation (nl MRI)	39	F/2600	*EPI*<br>(No BO)	No	No	PERT
		16	G542X/ F508del	ETI	28	25 gw→BH; BD(7); GB (nd)<br>28gw→BH; BD(5); GB (nd)	14	39	F/3400	*EPI*<br>(No BO)<br>↑GGT	No	No	PERT
		17	E822X/ F508del	ETI	29	Nl, preventative use	-	39	F/2390	*EPI*<br>(No BO)<br>↑GGT	Yes<br>2 months	No	PERT
		18	R334W/ F508del	ETI	26	Nl, preventative use	-	37	F/2980	*No EPI*<br>*No BO*<br>↑*Ub*	Yes	No	No PERT
		19	F508del/ G551D	IVA	32	Nl, preventative use	-	37	F/2650	*EPI*<br>(No BO)	No	No	PERT
		20	F508del/ c.2061_2062 ins	ETI	19	Nl, preventative use	-	39	F/3300	*EPI*<br>(No BO)	No	No	PERT
**Montironi R. et al. (the present case)**	1	21	F508del/ F508del	ETI	34	22 w→ mild BH, BD, GB (Doubtful presence)<br>33+2 gw→ single BD (11)<br>33+4 gw→ BD (22) with spiral course of the loops dilated<br>34+6 gw→ BD (30) with a spiral course of the dilated loops. Polyhydramnios.<br>35+5 gw→ BD (11-12). Meconium-filled cyst with a thick membrane of 7.5 x 5.5 x 4.6 cm	No resolution	37	M/ 2920	*BO*<br>(MI complcated by jejuno volvulus, perforation and meconium pseudocyst)<br>*EPI*<br>*Mild cholestasis*	No	Yes	Intestinal resection and terminal ileostomy 12h after birth<br>PERT from DOL 6<br>Ileo-ileal anastomosis (DOL 48)<br>FEF reached on DOL 60<br>Discharged home on DOL 63

GA, gestational age; W, weeks; GW, gestational weeks; Y, years; D, days; Sp, sample; n°, case number; ND, no data available; Nl, normal; NA, not administered; CF, cystic fibrosis; DOL, day of life; ETI, Elexacaftor/tezacaftor/ivacaftor; IVA : ivacaftor; US, ultrasound; CFTRm, CFTR modulator; MI, meconium ileus; M, male; F, female; BO, bowel obstruction; PERT, Pancreatic enzyme replacement therapy; EPI, Exocrine pancreatic insufficiency; MRI, magnetic resonance imaging; BH, Bowel hyperechogenicity; BD, bowel dilatation (mm of diameter); GB, gall bladder; RDS, respiratory distress syndrome; VTP, Voluntary Termination of Pregnancy; Ub, unconjugated hyperbilirubinemia; LPT, laparotomy; BW, Birthweight; FEF, full enteral feeding; NF, not found.
